# Simple foraging rules in competitive environments can generate socially structured populations

**DOI:** 10.1002/ece3.4061

**Published:** 2018-04-20

**Authors:** Mauricio Cantor, Damien R. Farine

**Affiliations:** ^1^ Departamento de Ecologia e Zoologia Universidade Federal de Santa Catarina Florianópolis Brazil; ^2^ Department of Collective Behaviour Max Planck Institute for Ornithology Konstanz Germany; ^3^ Chair of Biodiversity and Collective Behaviour Department of Biology University of Konstanz Konstanz Germany; ^4^ Edward Grey Institute for Ornithology Department of Zoology University of Oxford Oxford UK

**Keywords:** cooperation, foraging specialization, group dynamics, self‐organization, social network

## Abstract

Social vertebrates commonly form foraging groups whose members repeatedly interact with one another and are often genetically related. Many species also exhibit within‐population specializations, which can range from preferences to forage in particular areas through to specializing on the type of prey they catch. However, within‐population structure in foraging groups, behavioral homogeneity in foraging behavior, and relatedness could be outcomes of behavioral interactions rather than underlying drivers. We present a simple process by which grouping among foragers emerges and is maintained across generations. We introduce agent‐based models to investigate (1) whether a simple rule (keep foraging with the same individuals when you were successful) leads to stable social community structure, and (2) whether this structure is robust to demographic changes and becomes kin‐structured over time. We find the rapid emergence of kin‐structured populations and the presence of foraging groups that control, or specialize on, a particular food resource. This pattern is strongest in small populations, mirroring empirical observations. Our results suggest that group stability can emerge as a product of network self‐organization and, in doing so, may provide the necessary conditions for the evolution of more sophisticated processes, such as social learning. This taxonomically general social process has implications for our understanding of the links between population, genetic, and social structures.

## 1 INTRODUCTION

Animals face many challenges to stay alive. One taxonomically widespread solution is to live in groups. Individuals can increase fitness by reaping the benefits of associating with conspecifics, such as antipredator protection, movement energetic advantages, better access to mates, and knowledge about the environment (see Krause & Ruxton, 2002). A major axis of these benefits is accessing food. Groups can be more successful solving the problems of locating, capturing, handling prey (e.g., Clark & Mangel, 1986; Creel & Creel, 1995; Pitcher, Magurran, & Winfield, 1982). But, the benefits of group foraging are counterweighted by competition among group members reducing *per capita* food intake (e.g., Clark & Mangel, 1986; Creel & Creel, 1995; Packer, Scheel, & Pusey, 1990).

Ecological specialization is a tacit answer to competition (e.g., Futuyma & Moreno, 1988). The growing evidence for intrapopulational behavioral variation has challenged the postulate that species have a single foraging strategy (Kamil & Roitblat, 1985; Lang & Farine, 2017; Sargeant, 2007). If organisms opt for behavioral strategies that maximize their net energetic gains, this can motivate behavioral specializations that narrow individual niche breadth, increasing resource partitioning among individuals (Bolnick et al., 2003; Partridge & Green, 1985). Specialist foragers can target specific food items (e.g., Cantor, Pires, Longo, Guimarães, & Setz, 2013) that may be difficult to get without a certain morphological trait (Araújo et al., 2008) or learned technique (Ottoni & Izar, 2008; Sargeant & Mann, 2009). The literature is rich in examples of such specializations. In terrestrial species, communities of chimpanzees (*Pan troglodytes*) and orangutans (*Pongo borneo*) exhibit large repertoires of inventive foraging specializations (van Schaik et al., 2003; Whiten et al., 1999). At sea, the behavioral flexibility of marine mammals exemplify the case: Sea otters (*Enhydra lutris*) from different matrilines use tools to exploit distinct dietary items (Estes, Riedman, Staedler, Tinker, & Lyon, 2003); sympatric orca whale (*Orcinus orca*) pods have diverged into reproductively isolated ecotypes, specialized on hunting different prey (Baird, Abrams, & Dill, 1992; Beck, Kuningas, Esteban, & Foote, 2012; Ford et al., 1998), and populations of bottlenose dolphins (*Tursiops trucatus*) across the world contain social communities with remarkably diverse foraging strategies (see Whitehead & Rendell, 2014). However, while these examples demonstrate the outcome of hundreds or millions of years of evolution, how such specializations got underway remains less clear.

A number of recent studies on sociality have generated an important new insight: Many complex population‐level patterns of behaviors observed at the group or population level can be explained by simple processes of self‐organization (see Couzin & Krause, 2003; Sumpter, 2010). In the same vein, many apparently sophisticated behavior can be produced by simple psychological mechanisms (McAuliffe & Thornton, 2015; van der Post, Franz, & Laland, 2017; Raihani, 2015). Thus, it is important to explore simple mechanisms that could either explain or provide an evolutionary pathway to the widely observed patterns of structured foraging specialization. A key question is how could species make the jump from everyone foraging on the same resources to being partitioned into stable groups that forage on different resources. In many competitive environments, groups outperform individuals, but individuals within groups still compete for limited resource share. We hypothesize that in this scenario, a simple individual‐level rule—keep foraging with those you last foraged with if you acquired sufficient food—could lead to temporally stable subgrouping among individuals. We note that such a principle is simple enough to be followed by microbes (West, Diggle, Buckling, Gardner, & Griffins, 2007). This rule is interesting because decisions to engage in foraging with others generate a directed social network, and such networks can have global properties that emerge from the fact that many more edges (here associative foraging ties) are possible than the number of nodes (individuals) in the population. A final question is whether stability in group membership, and also which group forages at a given resource, can be maintained over generations. In other words, are foraging networks resilient to short‐term and long‐term stochastic events? If this is the case, then it would set the scene for diversification over evolutionary time.

We introduce an agent‐based model based on a static population to examine the essential conditions for the emergence of groups of specialized foragers. Then, we expand the model to include stochastic birth and death processes, and track stability in group structure across generations. We simulate an environment that contains a food patch requiring two or more individuals to be exploited. When an individual experiences a positive foraging outcome (i.e., the food patch contains an excess of resources relative to the number of foragers), then it seeks out additional foraging ties in order to increase its network size (i.e., the number of others with whom it is willing to engage in foraging behavior with). By contrast, if an individual experiences a negative foraging outcome (e.g., there were more foragers than what the resource can support), then the individual seeks to reduce its network size by randomly removing existing foraging ties. Our model investigates whether population‐level differentiation in resource use can emerge as a process of network self‐organization.

An empirical illustration of the fundamental process we model is the economic decisions of social predators. Picture a foraging individual chimpanzee, for example. Alone, it can survive off fruits, nuts, leaves, but when hunting in groups, it can help bringing down large mammals, such as Colobus (*Colobus* spp.) and Diana monkeys (*Cercopithecus diana*; Boesch & Boesch, 1989). Thus, when an individual decides to join a group to collectively access a better‐quality food source patch, it implicitly decides that the payoff of the hunting coordination will compensate for the cost of the food sharing system (Boesch, 2002). The payoff, of course, will depend on how much the resource yields relative to the size of the group it has to feed. In our models, this social dynamic is intentionally simplified to portray individuals who make social decisions (to increase or decrease the number of conspecifics they forage with) based only on their current experience, without planning for the future nor using any information about the global structure, past history of group membership, or how related they are to other individuals. Our simulations show that a simple process (“keep foraging with the same conspecific(s) if you were successful in accessing sufficient food last time”) is sufficient to form stable groups that completely control the focal resource. Thus, populations can easily exhibit basic resource‐use specialization—in this case structured groups that are maintained around one particular resource even in the absence of fitness costs or benefits associated with the resource.

## 2 MATERIALS AND METHODS

We simulate the emergence of foraging groups from simple interaction rules using an agent‐based modeling framework (Railsback & Grimm, 2012). Specifically, we investigate whether the propensity to forage with the same conspecifics if the last attempt was successful is sufficient to produce (a) populations exhibiting resource‐use specialization, and (b) structured patterns of relatedness among group members. We built two agent‐based models, a baseline model and a reproductive model, in R 3.2.0 (R Development Core Team, 2017) using functions from igraph (Csardi & Nepusz, 2006), sna (Butts, 2008), asnipe (Farine, 2013), and locfit (Loader, 2007) packages. All models, codes, and data are available at the online repository https://bitbucket.org/maucantor/coopgroup/src.

### 2.1 Baseline model

The purpose of the baseline model is to evaluate whether patterns of nonrandom social foraging on a focal patch, and thus socially structured populations, can emerge from individual social foraging rules. The model design is very simple. Individuals live in closed populations of size *N*, without birth, death, immigration, or emigration. The environment contains a patch one patch size *R* that is inaccessible to lone individuals. Exploiting this resource patch requires forming groups of two or more individuals. Individuals in the population can form associative foraging ties with any other individual (resulting in a directed binary network), where ties represent a willingness for that individual to forage with the other.

We initialize each simulation from a neutral starting point by randomly arranging directed foraging ties among individuals, with the probability that any given tie is present determined by the parameter *T*_prob_. Pairs of individuals, *i* and *j*, forage together if they have reciprocal ties (*T*_*i*,*j*_ = *T*_*j*,*i*_ = 1). Because an individual might have multiple reciprocal ties, we define groups as sets of individuals with reciprocal ties using a chain rule (empirical example: Smolker, Richards, Connor, & Pepper, 1992). That is, all individuals that are in the same connected component of the current reciprocation network are considered to form a group (e.g., A reciprocating with B and B reciprocating with C makes a group containing all three individuals, regardless of the links between A and C). However, while our group definition is transitive (e.g., A, B, C foraging on a patch at time *t* implies a connection between A and C via B), we do not update the network to make it transitive (although individuals can update their ties based on their experience, see below).

Individuals do not pay a cost if they do not forage on the patch. However, social foraging can entail a cost: When multiple groups are present, they compete to access the *R* units of resource. Because larger groups typically outcompete smaller groups (e.g., Schoener, 1971; Wrangham, 1980), we model the group‐level resource share as $s_{k}=R\cdot \left(n_{k}^{2}/\sum_{j=1}^{h}n_{j}^{2}\right)$, where the resource share *s* for group *k* of size *n*_*k*_ is determined by the size of the other *h* groups. We assume that the competitiveness of each group is determined by the square of the group size—following the same logic as the number of possible relationships in a group is proportional to the square of the size of the group. The group‐level resource *s*_*k*_ is then shared equally among group members. Thus, the expected *per capita* share of the reward *r*_*i*_ for an individual *i* is given by $r_{i}=s_{k}/n_{k}$. We briefly note that while this partitioning of resources first among groups and then among individuals reflects natural competitive processes, our model results are not sensitive to this rule—they provide qualitatively equal results based on a rule of equal allocation of resources among all individuals irrespective of group size ($r_{i}=R/\sum_{j=1}^{h}n_{j}$) (see Results; Supporting Information). We define the *per capita* share *r*_*i*_ as providing an optimal foraging outcome if *r*_*i*_ = 1, missed opportunities if *r*_*i*_ > 1, and a negative foraging outcome if *r*_*i*_ < 1. In our model, *n*_*k*_ tends to be restrained by the available resource *R*, meaning groups do not expand to include everyone; *n*_*k*_ also has a lower bound of *n*_*k*_ = 2, as the resource is not accessible to individuals.

We simulate repeated opportunities for groups to forage on this patch by repleting the resource in each time simulation step *t*. After each time step, each individual that foraged on the patch follows one of two rules to update their social ties: (1) when it experiences a negative foraging outcome (*r*_*i*_ < 1), meaning the resource share of the group is less food than the number of individuals requires, the individual randomly removes one of its outgoing ties, which can lead to decreased group sizes; (2) when it experiences a missed opportunity (*r*_*i*_ > 1), meaning the resource share of its group is greater than the number of individuals, it forms a new foraging tie with a random individual. This random tie could be either with another member of the same group (which would reinforce the foraging bond) or with another individual in the population (which can lead to a larger group size). That is, we allocate resources to individuals (i.e., calculate *r*_*i*_) only to inform the updating of ties, and the value itself contributes nothing else to the model outcomes. Instead, whether group sizes change depends on the new network structure generated by the updated reciprocated ties. In cases where the resource share of the group perfectly matches the number of individuals, then individuals’ ties remain unchanged. Individuals have no information about the consequences of adding or removing particular ties. The addition or removal of ties is random with respect to identity and group membership (within the limits that only existing ties can be removed, and only missing ties can be added). That is, we do not encode any rules for strengthening group structure. To model the process of random formation of new groups (e.g., for the purpose of competing with established groups and to test the robustness of group stability), in every time step there is a small chance (0.01%) of a tie being randomly added between a group member and any individual of the population.

In summary, our simulation has only three steps. First, we initialize a random network of ties among the individuals. Second, we allocate the resource based on the distribution of group sizes and share each groups’ allocation equally among its members (or share the resource equally among all foragers in groups, see Supporting Information). Finally, we allow individuals to update their propensity to engage with others in foraging together by adding or removing a directed tie at random. Steps 2 and 3 are repeated for 1,000 time steps in each simulation run.

Our simulation takes a resource‐centric approach; that is, we focus emergent group structure in a network that is shaped by foraging ties. By simply focusing on the associative ties that are formed or disbanded in the context of accessing a single beneficial resource type, we do not incorporate any information about the history of ties among individuals when determining their decisions. Thus, our model is Markovian, and we make no assumptions about memory, long‐term individual recognition, knowledge of global group structure, past rewards, preferred associations or social strategies (i.e., choosing more or less profitable ties).

Our simulation investigates how the foraging decisions of individuals with respect to a focal patch can generate a structured network of foraging ties. We suggest that the presence of one or more groups formed around exploiting the focal patch represents a simple case of a foraging specialization. In our models, this happens when a stable group exclusively uses the resource and outcompetes other individuals—noting that we model competition to generate alternative outcomes that could influence the stability in the network ties that underlie the formation of each group, and avoid making assumptions about energetics. In nature, exclusive access to a resource could or could not require specialized foraging techniques, but we do not explicitly model this. Instead, in our models the individuals that are part of a group mimic the process of specialization in the sense that by accessing the focal patch they (1) give up on using the other resource types available in the environment (see Futuyma & Moreno, 1988), and (2) their niche becomes narrower relative to the population's niche (see Bolnick et al., 2003). Individuals that are not part of a group survive by exploiting a variety of other alternative resource types, but we make no additional assumptions about their distribution or how they impact fitness. Finally, we note that our model can be equally considered as demonstrating emergent resource monopolisation by a group, but our motivation is to understand whether this process of monopolisation could arise in the broader context of it underpinning ecological specialization.

### 2.2 Reproductive model

While our baseline model implements interactions among individuals in a fixed population, the purpose of our reproductive model is to investigate whether self‐organized structured networks of foraging ties are robust to demographic processes. By including births and deaths—that were stochastic, occurred at a constant rate, and independently of the energetic rewards—the reproductive model evaluates whether interactions associated with access to food resources can be maintained over generations. This also allows us to investigate whether kin‐structured groups are formed in the resulting network. This model used the same design as the baseline model, with two additional steps.

First, at each time step individuals can reproduce. Reproduction is sexual, and we assume that any individual in the model can reproduce. To avoid complications with assumptions about mate formation, we model a population containing only a single sex (in our case females given that we were interested in patterns observed mostly in vertebrates). We therefore assume that females reproduce with random uncounted males, produce a single offspring, and do not emigrate; males, on the other hand, always disperse and never engage in the formation of groups. In our model, the probability that an individual of a given age (*a*, the number of time steps it has been alive) has a (female) offspring in a given time step *t* is $P_{r}\left(a\right)=10^{-3}\cdot \left(\frac{1}{1+e^{\left(-0.1a+5\right)}}\right)$. That is, we use a sigmoidal function to model individuals becoming more likely to reproduce as they become older (Clutton‐Brock, 1984) and therefore match the death rates (see below).

When an individual reproduces, its offspring inherits all of the parent's ties (both incoming and outgoing). The offspring also create a tie with the parent, but this tie is not reciprocated (to avoid forming a new group each time an offspring is born). This means that if the parent is already part of a group, the offspring will be part of the group as it has all of the reciprocal ties that the parent has. At this point, the offspring is subjected to the same group dynamics of the baseline model, as all other group members, including its parent. An important consequence of this is that the *per capita* share *r*_*i*_ for each individual in this group will decrease as a result of increased group size. Thus, if *r*_*i*_ < 1 at time step *t *+* *1, which will be the case if the group size was optimal prior to the birth, then all members will randomly remove an existing tie. This mimics the process in which a birth into a group will reduce the overall resource pool available to everyone else. As a result of tie removals, either or both the parent and the offspring can be kept in the group or not, and this process is stochastic. We do not encode any rules promoting natal philopatry or parental care, neither do we explicitly encode long‐term kinship recognition (in fact our model discourages it as we do not form a reciprocated tie with the parent). Instead, we model a situation in which an individual is born into the same social space as its parent, from which point it is treated as any other individual.

Second, individuals can die, upon which all of their associates lose one tie. The probability for an individual dying is given as *P*_*d*_(*a*) = *P*_*r*_(*a*). That is, individuals reproduce and die at the same rate so that the overall population do not grow unboundedly: Its size remains relatively stable, but can vary from time step to time step as births and deaths are stochastic.

In brief, our reproductive model contains the same three steps as our baseline model, with the addition of reproduction and death occurring at each time step. Each simulation is initialized with populations containing completely unrelated individuals. We emphasize that we do not encode any rules encouraging individuals to form or maintain reciprocated ties with kin, as there is no kin recognition.

### 2.3 Metrics

For the simulations using the baseline model, we measure five properties at each time step: (a) number of groups, (b) group size, (c) individual payoffs, (d) social stability, and (e) exclusivity. Groups (a) are transitive, formed via the chain rule explained above. Group size (b) is measured as the average number of individuals in all groups (excluding *n*_*k*_ = 1, i.e., lone individuals, as the minimum group size to access the resource *R* is 2). Individual payoff (c) is calculated as the mean *per capita* share *r*_*i*_ across all individuals where *r*_*i*_ > 0 (i.e., only those that are in a group that gain access to the resource patch). Social stability (d) is defined as an undirected network representing the proportion of all time steps that two individuals have a reciprocal tie (i.e., the stability of individuals *i* and *j* at time *t* is given by $s_{i,j}\left(t\right)=\frac{\sum_{x=1}^{t}\left(T_{\mathrm{ijx}}=T_{\mathrm{jix}}=1\right)}{t}$). Finally, we define a measure of group‐level stability called exclusivity (e). Exclusivity represents the extent to which a single, optimally sized, group of individuals exploits the resource. In network terms, exclusivity is defined as the proportion of the total edge weights (proportion of network density) in the social stability network ($E=\sum s_{\mathrm{ij}}$) that is accounted for by the *R* individuals with the highest degree (those with the most, or most consistent, reciprocal ties over time). An exclusivity value of *E* ≈ 1 indicates that an optimal group of size *R* exclusively exploits the resource for most of the time. That is, one group becomes specialized on the resources and monopolizes the resources it contains. Low values of exclusivity indicate an unstable social structure with frequent group membership turnover or infrequent optimal group sizes.

For the simulations using the reproductive model, we also record (f) pedigree, (g) relatedness, (h) average relatedness among members of each foraging group (hereafter “members” as opposed to “nonmembers”), and (i) log‐ratio of the relatedness among members versus nonmembers, and the value of this relatedness expected by chance. Pedigree (f) is defined as a network where edges represent parent–offspring relationships. Relatedness (g) is defined as a network where edge weights are calculated based on the pedigree relationships using $l_{\mathrm{ij}}=1/2^{d_{\mathrm{ij}}}$, where *l*_*ij*_ is the relatedness between individuals *i* and *j*, and *d*_*ij*_ is the shortest path length connecting the individuals *i* and *j* (*l*_*ij*_ is set to 0 if they are not connected). Thus, as we assume sexual reproduction in diploid eukaryotes without inbreeding, relatedness between a parent and its offspring (and between full sibs) is 0.5, between a grandparent and grand‐offspring (and half‐sibs) is 0.25 etc. The average relatedness (h) among members (*l*_*m*_) is calculated for each time step by first averaging the relatedness among members of each foraging group, then averaging the relatedness among groups. The log‐relatedness ratio (i) is calculated by dividing the average relatedness among members by the relatedness expected by chance: *L*_*t*_ = log(*l*_*m*,*t*_/*l*_*r*,*t*_), where *L*_*t*_ is the natural logarithm of the relatedness ratio at the time step *t*;* l*_*m*,*t*_ is the average relatedness among members at the time step *t*; and *l*_*r*,*t*_ is the relatedness expected by chance at the time step *t*.

We use a permutation test to calculate the relatedness expected by chance (*l*_*r*,*t*_). This step is necessary given that underlying relatedness varies stochastically across time steps and across different replicates of the model (and starts at 0). Our permutation test is simple. At a given time step *t*, we first randomly create the same number and size of groups by resampling all individuals currently alive with equal probability and without replacement; then, we calculate their relatedness as carried out for *l*_*m*_; and, finally, we repeat steps 1 and 2 for 100 times and calculate the average relatedness based on these 100 possible sets of groups drawn from the current population.

### 2.4 Sensitivity analysis

We perform a sensitivity analysis to evaluate the consistency of the outputs of simulations and their robustness to variation in initial conditions. We simulate the baseline and the reproductive models across a wide initial parameter space, defined by varying initial population size *N* by increments of 5 (P={2,7,12,…,200},N∈P); varying resource patch size *R* by increments of 2 (S={5,7,9,…,51},R∈S); and varying the random initial social network connectivity *T*_prob_ (i.e., proportion of realized links) ($C=\left\{0.2,0.5,0.8\right\},\;T_{\mathrm{prob}}\in C$). For each parameter combination, we generate 500 replicates of the baseline model and run them for *t* = *N* · 5 time steps (T={100,…,1,000},t∈T), which is typically about 10 times more steps than is required for groups to emerge and stabilize (adding more steps does not change the results). For the reproductive model, we generate 100 replicates and run each for 1,000 time steps, which is enough time to allow for demographic processes to operate across multiple generations (typically 4.5, calculated as the diameter of the full pedigree network at the end of the simulation, averaged across the 100 model replicates). We consider the entire parameter space when evaluating exclusivity (*E*) and log‐ratio of relatedness (*L*_*t*_). For all other metrics, we consider four representative areas of the parameter space: small population size and small resource patch size; small population and large patch sizes; large population and large patch; large population and small patch.

## 3 RESULTS

Our models rooted on basic group dynamics reiterate that the rewards of accessing and the costs of sharing food contribute to optimal group sizes. We are careful to make our models as simple as possible. The three only rules implemented in our baseline model are as follows: (1) Groups can access a high‐value resource, whereas individuals cannot; (2) larger groups get a bigger share and individual share is proportional to group size; (3) group members add or remove associative foraging ties based on the share of resources they experience at the current time. For the reproductive model, we make two additional assumptions: (1) Births and deaths are stochastic, relatively constant in rate, and occur at the same rates to maintain a generally constant population size; and (2) newborns receive their parent's associative foraging ties at their birth, but are immediately subjected to the stochastic group dynamics based on individual payoffs.

### 3.1 Baseline model: emergent stable foraging groups in closed populations

**Figure 1:**
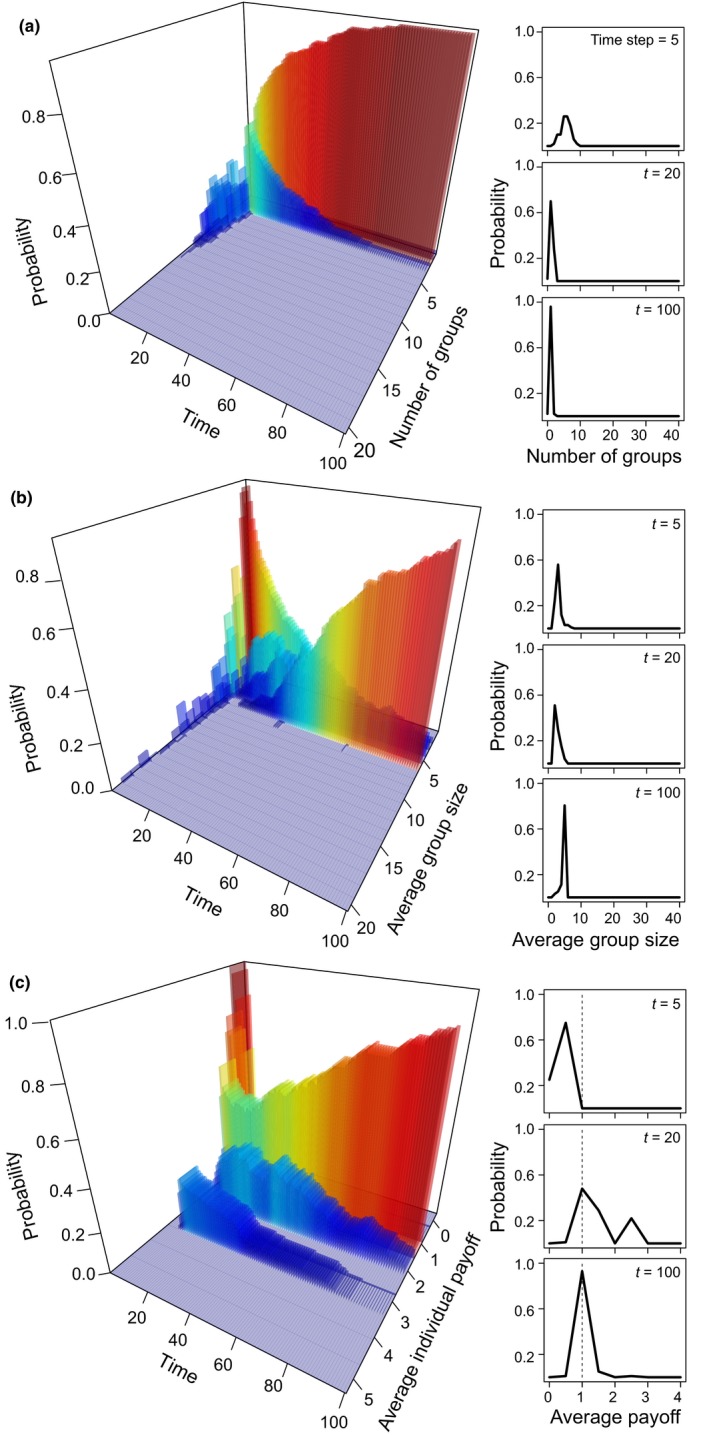
Emergent properties of simple social foraging rules. The (a) number of foraging groups, (b) average group size, and (c) average individual payoff, rapidly stabilized over time, resulting in a single group of size *R* where each individual receives an optimal payoff. Probability distributions are calculated as the observed value divided by the total value of the metric at each time step *t*. Inset plots represent three snapshots of the simulation time (vertical dashed lines in (c) indicate the optimal payoff *r*_*i*_ = 1). The simulations were run for 500 replicates of the baseline model with population size *N *=* *40, resource patch size *R *=* *5, initial connectivity among nodes *T*_prob_ = 0.2, and 200 simulated years (time steps = N*5, but for clearer visualization, the plots were truncated at time step *t *=* *100). These patterns are consistent with other areas of the parameter space (Figure S1)

**Figure 2:**
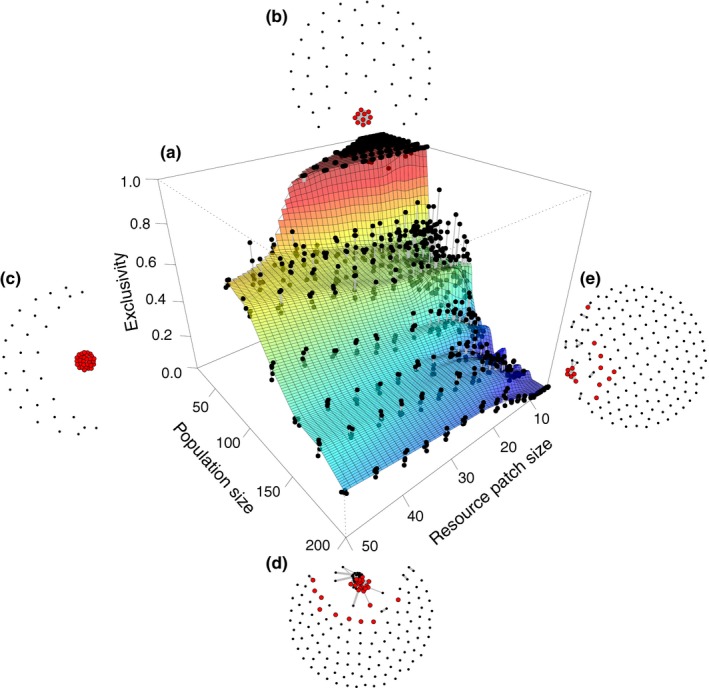
The emergence of foraging groups in the baseline model. In the surface plot (a), exclusivity (proportion of network density among the top connected individuals) is plotted as a function of initial population sizes (i.e., number of nodes in the network, *N*) and resource patch sizes (*R*). Each point is the result of one replicate of the simulation. In the social networks representative of four areas of the parameter space (b–e), individuals (nodes) are connected by the proportion of times (links) they are part of a group (links <0.3 are filtered out for better visualization, but included in all analyses). Red nodes indicate members of the emergent specialized foraging group at the end of the simulation. Data are based on 500 replicates of the model for each combination of *N* and *R* parameters. The initial network connectivity is *T*_prob_ = 0.2 (i.e., ~20% of possible links are randomly assigned when the network is initialized), but the results are independent of initial connectivity values (Figure S2)

Our baseline model investigates the links between population size, resource patch size, and the emergence of stable foraging groups. When population size is small, groups of individuals initially compete for the resource patch (Figure 1a, time step *t *=* *5), but rapidly a single, optimally sized group (containing *R* individuals, where *R* is equal to the resource size) with stable membership emerges. In other words, *R* individuals have become specialized by exclusively exploiting this resource type (Figure 1, time step *t *=* *100), and by monopolising it, and they have a nonoverlapping resource use relative to all other individuals in the population. However, the emergence of a stable group typically decreases with a combination of increasing population and resource patch sizes (Figures 2, S1 and S2). A sensitivity analysis indicates that these results are highly consistent across a range of initial network connectivity (Figures S1 and S2a–c) and robust to changes in the type of group‐level resource share, that is, whether the allocation of resources is dependent on group size or not. When the resource is equally allocated among all individuals irrespective of group size, a single stable group also emerges in small populations and monopolizes the small resource patches (Figure S2d). Although under the scenario of equal allocation of the resource, the emergence of specialization seems at first sensitive to higher network connectivities (Figure S2e,f), we note that it simply takes longer to establish (Figure S2g–i).

When small populations live in an environment containing a small patch of a high‐value resource, the propensity for the patch to be monopolized by a single group that becomes specialized on that resource type (exclusivity) is particularly high (*E* ≈ 1; Figure 2b,c). In this situation, within just a few time steps, a single and stable group is formed (Figure 1a), of optimal size (e.g., number of individuals equal to the resource patch size; Figure 1b), in which individuals receive an average of 1 resource unit each (our modeled optimal value, Figure 1c). By contrast, when the resource patch is large (Figure 2d), multiple and unstable groups of varying sizes are formed (Figure S1c). When the patch is small, but populations are large (Figure 2e), fewer groups are formed and these do not stabilize at the optimal group size (as shown by higher variance in average individual payoffs; Figure S1d).

Our baseline model demonstrates that a simple rule in which individuals continue to forage with the same conspecifics with whom they were successful before, and increasing or decreasing their pool of foraging associates based on the resource share they recently experienced, can lead to the rapid and consistent emergence of a stable group based around a resource. Hence, very simple resource specialization can arise without the need for fitness differentials, or any kind of long‐term memory.

### 3.2 Reproductive model: emergent within‐group relatedness and stable social structure across generations

**Figure 3:**
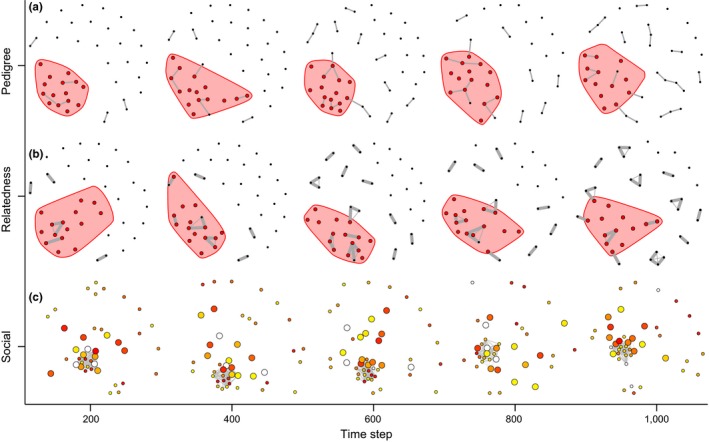
Evolution of pedigree, relatedness, and social relationships in the reproductive model. Simulations were run for the parameter space area representing small population and resource patch sizes (see Figures 2b and 4b). The pedigree networks (a), showing all individuals that are alive at a given time step (*x*‐axis), show that related individuals (nodes connected by links) are frequently found in foraging groups. Red nodes (and shading) indicate individuals currently part of the single foraging group. Similarly, the relatedness networks (b) show that individuals within social groups are often highly related (the thicknesses of links are proportional to their relatedness). However, the network depicting individuals are connected by the proportion of times they have been part of a specialized foraging group (c) suggests that foraging groups often contain individuals from different genetic lineages (here node color represents unique genetic lineages; for better visualization links whose weights <0.3 are filtered out). The simulations are based on a population size *N *=* *40, resource patch size *R *=* *15, initial connectivity *T*_prob_ = 0.2, and run for 1,000 time steps, and these patterns are consistent across the parameter space (Figure S3)

**Figure 4:**
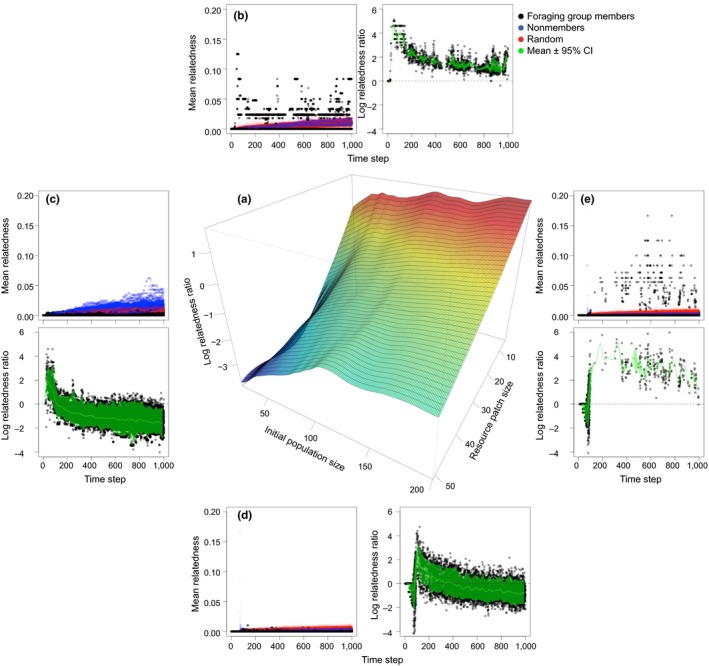
Relatedness among members and nonmembers of emergent specialized foraging groups. Environments with a smaller resource patch lead to greater relatedness among group members, suggesting that the more specialized a population, the more related individuals are simply by chance. The surface plot (a) displays the log of the ratio between the relatedness among members of the emergent foraging group (“members”) and the average relatedness among all other individuals (“nonmembers”) at the end of the simulation (time step *t *=* *1,000) as a function of initial population size *N* and resource patch size *R*. Positive log‐ratio indicates higher relatedness among members than nonmembers. The scatterplots (b–e) display relatedness outputs of 100 model replicates throughout the entire simulation (1,000 time steps each), representative of the four areas of the parameter space. In the colored scatterplots, black circles represent the average relatedness among foraging group members; blue circles represent average relatedness among nonmembers; and red circles represent the average relatedness among 100 randomly chosen sets of individuals of the same size of the foraging groups. In the other scatterplots, black circles represent the log of the relatedness ratio between foraging group members and sets of randomly chosen individuals throughout the simulation time for 100 model replicates. Here, positive log‐ratio indicates higher relatedness among members than expected by chance. Green lines represent the mean log‐ratio across model replicates, and green polygons indicate 95% confidence intervals. Horizontal dashed lines indicate log‐ratio = 0. Cases of undefined relatedness ratio (*l*_*m*,*t*_ = *l*_*r*,*t*_ = 0 or *l*_*r*,*t*_ = 0) and zeroed ratio (*l*_*m*,*t*_ = 0 → ln(0) = −∞), making the plots for small population sparser (b,c). In all cases, the initial network connectivity is *T*_prob_ = 0.5. Each combination of *N* and *R* parameters is run for 100 model replicates, and each model is run for 1,000 time steps

Ultimately, we aim to explain population structure sustained over generations. Our reproductive model extends the baseline model by including birth and death processes, tracking both the population structure as well as the pedigree and relatedness among group members and nonmembers. As simulation time progresses, we find that the network of foraging ties, and the resulting population structure, is robust to the fluctuations caused by the addition of group members. Although foraging groups often contain individuals from multiple genetic lineages (Figure 3), the relatedness among members is generally higher than expected by chance (Figure 4a) because individuals are born into natal groups, and membership of these groups is relatively stable. The ratio of relatedness within groups (i.e., the log of the average relatedness among group members divided by the relatedness expected by chance) tends to decrease as time steps progresses (Figure 4). However, there is a clear tendency for relatedness to be mapped onto the formation of emergent foraging groups. This tendency is stronger when resource patch size is small (Figure 4b,e) than when patch size is large (Figure 4c,d), and is only weakly affected by initial population size. A sensitivity analysis shows that these findings are independent of initial conditions (Figures S3 and S4).

## 4 DISCUSSION

Our models reveal that a simple foraging rule for socially exploiting a resource can lead to the maintenance of stable foraging groups. When group members can survive exclusively from this resource, and by monopolising it against other competing groups, the emergent group structure results in a simple case of resource specialization—that is, group members give up other resources and have a narrower niche. Further, it could be argued that they also have specialized foraging behavior—group foraging. The networks formed by reciprocal foraging ties are also robust to disturbances, such as the random formation of new groups, the birth of new individuals into the group, and the deaths of group members. The relatively stable social structure is likely to be maintained as a result of the underlying network of foraging ties. This long‐term stability means that if offspring born into these groups are admitted to the same social bonds as their parents, then the system can easily exhibit kin structure in the social group. Our finding implies that the foundations of a social system in which foraging specializations are present and in which groups are kin‐structured could easily arise as an emergent phenomenon of simple individual‐level rules based around resource exploitation (here, forage with those with whom you were last successful). The following two principal outcomes of our models demonstrate how the maintenance of social ties based on direct foraging benefits can lead to a group structure that mirrors at least the baseline structure of real‐world populations (e.g., Estes et al., 2003; Kopps et al., 2014; Krützen et al., 2005; Wright, Stredulinsky, Ellis, & Ford, 2016).

First, competition for food resources alone can produce stable groupings and structure that is maintained over generations. As an inescapable cost of social life, competition is a central factor shaping the structure and dynamics of animal communities and is generally considered to reduce the propensity to be social. However, when groups are more efficient at exploiting a resource, such as in social predators (Lang & Farine, 2017), this creates situations in which groups must compete for that resource, and such competition can act to promote sociality. For instance, it has been suggested that female‐bonded grouping in several primate species can be driven by competition if within‐group cooperative foraging ties grant better access to high‐quality, but finite food patches (Wrangham, 1980). Our model shows that this can be true even without any differential energetic outcomes among groups—but can instead emerge from the fact that larger groups have many more potential reciprocal ties that makes them more robust to short‐term stochastic events. These findings are consistent with recent evidence that resource competition can promote group territoriality (Port, Schulke, & Ostner, 2017) and from game‐theoretical models that suggest cooperative group foraging can be a consequence of predator gregariousness when, under certain conditions of prey availability, it outweighs the advantages of foraging alone (Packer & Ruttan, 1988). We advance the more general prediction that groups should become more specialized on a resource, which could result in territoriality (i.e., monopolising a spatial area) but could also result in foraging niche divergence. Further, our baseline model suggests that small population size is one key condition for stable groups, and thus specialization, to emerge. In our simulations, despite varying initial conditions, increasing population and resource patch sizes generally promote multiple foraging groups with unstable membership. Thus, our model predicts that stable groups of optimal size should be more likely in relatively small populations, especially those experiencing high levels of competition for scarce and patchy resources.

Second, after multiple generations, members of the same foraging group were more genetically related than expected by chance. Across all initial conditions, our reproductive model generated foraging groups that typically contained more than one genetic lineage. However, the relatedness ratio between members and nonmembers tends to decrease over time. This probably happens due to more births occurring outside of groups than within groups (i.e., simply because there are more individuals outside of groups). We highlight that, once under the conditions of living in stable foraging groups, within‐group genetic relatedness could naturally increase through alternative routes not encoded in our models. The first is via fitness benefits. If access to the resource increases individual fitness, there would be a higher birth rate within versus between groups. Such fitness benefits would generally work to enhance all of the patterns we present (e.g., the stability of groups over time). The second is via kin recognition or kin discrimination (Mateo, 2004; Wright et al., 2016). If individuals have developed the ability to recognize and preferentially associate with kin, groups of foragers could easily become composed of relatives. The final route is via natal philopatry and parental care (Wilson, 1975). If mother–offspring bonds have not been subjected to the stochastic dynamics based on individual payoffs that can keep or expel other individuals from the group, relatives would tend to be more aggregated through time. In our model, we intentionally do not assume (or encode) any of these processes beyond the point at which the offspring is born into existing groups, and yet foraging groups can maintain high relatedness among group members based simply on the maintenance of foraging ties that offspring inherit (without needing a reciprocal tie from their mother). Their stable co‐associations across time could be a precursor allowing these more sophisticate (and potentially more reliable) mechanisms to evolve.

### 4.1 Ripple effects of associative foraging ties

While our models do not require the more complex aforesaid processes, we hypothesize that a similar emergent process could have facilitated their evolution. Our models show that socially stable groups with foraging specializations can emerge and persist without complex rules of group formation or cognitively demanding learning processes (see also van der Post et al., 2017), but instead as a result of networks of foraging ties that are shaped by recent individual experiences. The future strategies based only on the current state of individuals’ associative ties provide the initial opportunity for individuals to stay together with whom they had foraged successfully. In the face of competition from others, larger groups can ride out short‐term negative experiences (i.e., removal of foraging ties) because they have more connections available. That is, for a group of size *n*, there are *n*·(*n* − 1) possible ties. If the group experiences too few resource units and members shed ties (i.e., removing a maximum of *n* ties), then there remains a possible *n*·(*n* − 2) ties. The impact of removing *n* ties will therefore be much larger for small groups than for large groups: For *n* = 2, no ties might remain, for *n* = 3 as few as 50% of ties might remain, whereas for *n* = 11, a maximum of 10% of ties would be removed.

The stability provided by simple networks of foraging ties might then have allowed more complex mechanisms to develop in real‐world animal societies. In this situation, it would be advantageous for individuals to remember who they are most successful with (individual recognition), to track who their kin are (kin recognition), or for offspring to remain in their group (natal philopatry) to try and profit from food provisioning (McNamara & Houston, 1997) or reap other nonforaging benefits such as parental care. Any of these cases could set the stage for preferential associations toward these individuals to be selected for. From foraging benefits motivating related individuals to stay in groups, further differentiation—behavioral, cultural, genetic—could be promoted through time. Stable groupings, which here we propose could be driven by competition for limited resources alone, then provide favorable contexts for individuals to copy behavior from one another (e.g., Estebán et al., 2016; Klopfer, 1961; Whitehead & Rendell, 2014), promoting between‐group behavioral divergence that can be reinforced by cultural or genetic drift and selection (e.g., Cantor & Whitehead, 2013; Filatova et al., 2015).

### 4.2 But what about the real world?

Generally, specialized foraging groups occur in small wild populations, as seen in communities of primates, birds, cetaceans with foraging traditions shared by a subset of individuals (e.g., Aplin et al., 2015; Estes et al., 2003; Lamon, Neumann, Gruber, & Zuberbühler, 2017; Mann, Stanton, Patterson, Bienenstock, & Singh, 2012; Ottoni & Izar, 2008; van Schaik et al., 2003; Whitehead & Rendell, 2014; Whiten et al., 1999), and as predicted by our model. Further, members of communities of specialized foragers tend to be genetically related (e.g., Estebán et al., 2016; Estes et al., 2003; Kopps et al., 2014; Krützen et al., 2005; Lamon et al., 2017; Whitehead, 1998). A well‐documented illustrative case is the remarkable differentiation among ecologically distinct killer whales (see de Bruyn, Tosh, & Terauds, 2013). Social communities composed by groups of related individuals, even in sympatry, differ in a variety of behavioral and morphological traits, which seems to be rooted on dietary specialization. By specializing on distinct prey—most famously mammals versus fish (Ford et al., 1998)—killer whale social communities display distinct movement and hunting techniques more adjusted to their particular feeding habits and surrounding environment, along with distinct social systems and communication repertoires (e.g., Filatova et al., 2015; Yurk, Barrett‐Lennard, Ford, & Matkin, 2002). Finally, from their matrifocal social structures, foraging specializations seem to have triggered not only behavioral, but also genetic divergence (Foote et al., 2016; Hoelzel, Dahlheim, & Stern, 1998).

Our model highlights that initial dietary specializations could have been driven by competition and not necessarily only by innovation. There is growing empirical evidence that food availability and clumpiness can drive flexibility in social and foraging ties of marine predators (e.g., Gazda, Iyer, Killingback, Connor, & Brault, 2015), even in the stable social tiers of killer whales. Type, biomass, predictability, and density of prey influence not only foraging strategies but also social strength and connectivity, suggesting that ecological conditions can bend even phylogenetic inertia (Beck et al., 2012; Foster et al., 2012; Tavares, Samarra, & Miller, 2016). Our model captures that initial period—all individuals were the same, but from these emergent cliques there could arise increasing behavioral differences through time. Our model underlines how simple this initial process could have been.

Further empirical evidence for the initial emergence of foraging groups can also be drawn from studies of specializations on anthropogenic resources. Examples of groups of social animals exploiting human‐derived food patches abound: Bears exploit garbage (Mccarthy & Seavoy, 1994), and elephants and chimpanzees depredate crops (Chiyo, Moss, & Alberts, 2012; Hockings, Anderson, & Matsuzawa, 2012), while dolphins associate with aquaculture farming (Díaz‐López & Shirai, 2008), beg food from anglers (Donaldson, Finn, Bejder, Lusseau, & Calver, 2012; Powell & Wells, 2011), and interact with artisanal and commercial fisheries (Daura‐Jorge, Cantor, Ingram, Lusseau, & Simões‐Lopes, 2012; Kovacs, Perrtree, & Cox, 2017). Two particular cases of cetaceans foraging around fisheries indicate how the initial motivation to lessen intrapopulation competition by specializing on a resource can also have social consequences, in line with the predictions of our model. First are bottlenose dolphins that specialized on feeding around trawler vessels (e.g., Ansmann, Parra, Chilvers, & Lanyon, 2012). During the 1990's prawn trawling activities in Moreton Bay, Australia, some individuals formed an exclusive group that followed vessels to forage on discarded trawl bycatch. These dolphins essentially ate the same type of food as others in their population. As a result of specializing on a particular source of food, these individuals rarely associate with the other individuals from the wider population. However, this social distinction dissolved upon the closure of that fishing activity in the area: A decade after, members of the trawler group were completely mixed back into the broader population (e.g., Ansmann et al., 2012).

The second case are, again, the killer whales. In the Strait of Gibraltar, killer whales normally hunt bluefin tuna (*Thunnus thynnus*) by chasing them to the exhaustion. Since mid‐1990s, some individuals started to depredate the tuna fishery and specialize on raiding tuna from droplines (Estebán et al., 2016). The lower energetic costs (i.e., limited movement around boats, and not chasing prey) made this specialization so rewarding that new individuals were recruited into the group of depredators—until a certain point. The original group, which was characterized by homogeneous and strong associations, may have reached its carrying capacity and split into two less cohesive groups of depredators (Estebán et al., 2016). The conceptual construct of our model implicitly embrace the social plasticity demonstrated by these two examples. In many situations, the reward of a foraging specialization depends on the number of individuals exploiting a given resource (e.g., Estes et al., 2003; Svanbäck & Persson, 2004).

### 4.3 Caveats

Our models are purposefully simplistic. We aim to capture simple elements of ecology that could, at least partly, explain patterns in nature that are generally ascribed to complex social behavior. Thus, our models align with the recent motivation of switching from the description of patterns to their generative processes (Ilany & Akcay, 2016a,b). We are motivated by developing an understanding of resource specialization in vertebrates, but our model could equally be used to simply explore a process of emergent resource monopolisation. However, by extending the model beyond a single pool of individuals into multiple generations, we demonstrate that the conditions from which resource specialization could arise are relatively simple to develop. From this point, there are likely to be other factors, such as mate choice (we model only a single sex) and parental care (individuals in our model are faced with the same stochastic group membership from birth), that could also promote individuals to maintain groups.

Our model does not account for well‐known factors that impact foraging behavior, such as physiology, energetics, perception, fitness consequences, ranging area, learning, or time budgets (e.g., Clark & Mangel, 1986; Kamil & Roitblat, 1985; Pollard & Blumstein, 2008; Schoener, 1971). Nor do we make major assumptions about the resources such as renewal rates or quality. Instead, our model is inspired by models from studies of collective behavior, where the structure of the population emerges from the patterns of interactions among individuals (Couzin & Krause, 2003; Sumpter, 2010). We believe that many within‐individual factors would enhance the strength of our findings. For example, individuals are likely to expend less energy foraging in groups than foraging alone (e.g., through higher efficiency due to shared vigilance or through reduced searching time), which would increase the rates of formation (or the tendency for maintenance) of foraging ties in larger groups. By contrast, unsuccessful foraging from the resource patch could represent an opportunity cost, which could have severe impacts on fitness. We felt this assumption was justified because we were motivated by empirical studies on large and long‐lived group‐living animals such as primates and cetaceans that are likely to be able to overcome short‐term fluctuations in resource consumption. However, should insufficient *per capita* resource shares have significant impact on organisms, it could lead to rapid destabilization of group structure (e.g., if member shed many ties, or members are lost due to starvation) and could represent a major difference in the evolutionary pathways of group emergence across different types of taxa. This conclusion is supported by our lack of evidence of resource specialization in large populations, thus also restricting our proposed mechanism to typically larger and rarer species. Although simplistic, our models reinforce the powerful effects of emergent self‐organization in foraging networks where ties are based on direct individual experience, and propose the potential role of these effects as a precursor of social patterns and more sophisticate social processes.

## 5 CONCLUSIONS

We have demonstrated how a simple rule for foraging interactions can give rise to stable social groups. In small populations with limited resources, this can drive the emergence of specialized foraging groups consisting of related individuals. Our model captures many features of vertebrate social species that have been observed in the real world. For example, populations containing groups that forage on distinct resources are typically observed in small populations. Because such social communities are mostly found in species with high ability for cognition, such as social carnivores, apes, cetaceans, birds, they have typically been ascribed to sophisticated processes such as social transmission of behavioral traits and kin selection. Our simulations suggest that many of the features of these societies—such as stable, kin‐structured, and specialized foraging groups—can emerge or be maintained from simple individual‐level rules based on direct benefits, acquired via group foraging and stochastic demographic process in the face of competition for resources. We do not claim that the taxa we have focused on lack the cognitive or communication skills to develop local traditions via social learning or that inclusive fitness is not a contributing mechanism of this group structure. Instead, our models take us back to one basic stimulus for grouping—energetic reward—as a fundamental mechanism underpinning kin‐structured and specialized foraging groups.

In nature—we posit—immediate benefits of associative foraging ties could have given rise to initial social structure, as has been observed in several taxa, subsequently allowing more sophisticated processes leading to behavioral, cultural, or genetic differentiation to operate upon the stable co‐associations of individuals. One key challenge facing empirical studies aimed to explain how populations can be structured into groups with distinct behavioral repertoires is to unmask or account for the simple social dynamics rooted on competition herein reported, to then show how far social learning and cooperation toward kin are likely to have taken place. Doing so will improve the interpretation of the interplay between genetic and social structure, and thus advance our understanding of social evolution.

## CONFLICT OF INTEREST

None declared.

## AUTHOR CONTRIBUTIONS

DRF conceived of the study, programmed the core of models, helped writing the manuscript, and supervised the study. MC developed the complete models, ran simulations, analyzed data, prepared results and figures, and drafted the initial manuscripts. Both authors gave final approval for publication.
