# Supplementary material for: Simple foraging rules in competitive environments can generate socially structured populations
Source: Ecol Evol. 2018 Apr 20;8(10):4978–91. doi: 10.1002/ece3.4061 (PMC5980395; doi:10.1002/ece3.4061)
Supplement: Supplementary file 1 [file ECE3-8-4978-s001.docx]

Electronic Supplementary Material for the article:

Simple foraging rules in competitive environments can generate socially-structured populations

Mauricio Cantor^1^*, Damien R. Farine^2,3,4^*

^1^ Departamento de Ecologia e Zoologia, Universidade Federal de Santa Catarina, Brazil.

^2^ Department of Collective Behaviour, Max Planck Institute for Ornithology, Germany.

^3^ Chair of Collective Behaviour and Biodiversity, Department of Biology, University of Konstanz, Germany.

^4^ Edward Grey Institute of Field Ornithology, Department of Zoology, University of Oxford, United Kingdom.

* Contact: m.cantor@ymail.com; dfarine@orn.mpg.de

**
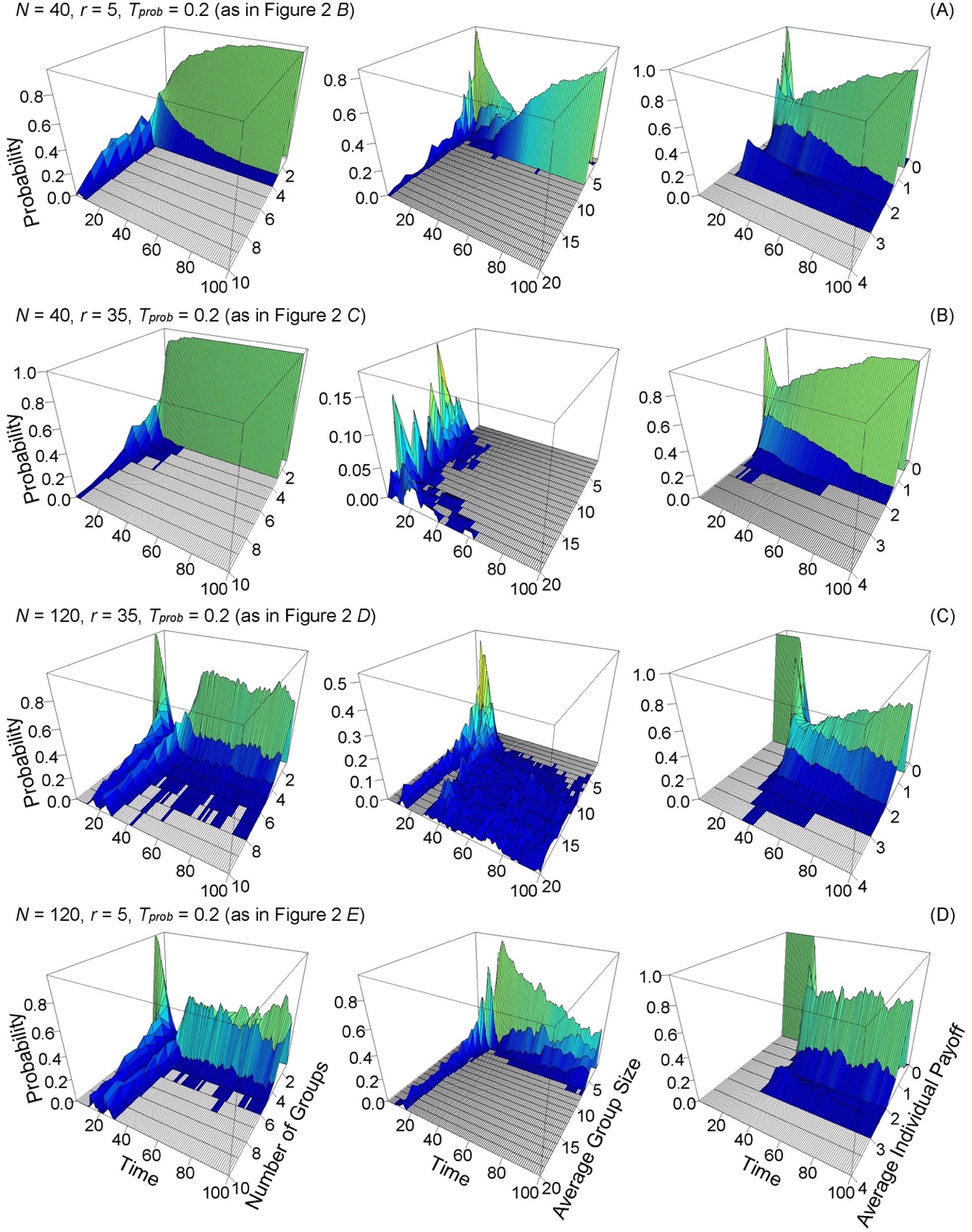
**

**Supplementary Figure S1:** Emergent number of specialised foraging groups, average group size, and average individual payoff in the four representative areas of the parameter space (see Fig. 2B-E): (A) small population size, small resource patch size; (B) small population, large patch size; (C) large population, large patch size; (D) large population, small patch size. *N* = initial population size; *R* = resource patch size; *T_prob_*=initial network connectivity. Simulations lasted for *N*5* time steps, but for better visualization, plots were truncated at time step *t*=100. Plots contain 500 replicates of the model for each parameter combination.


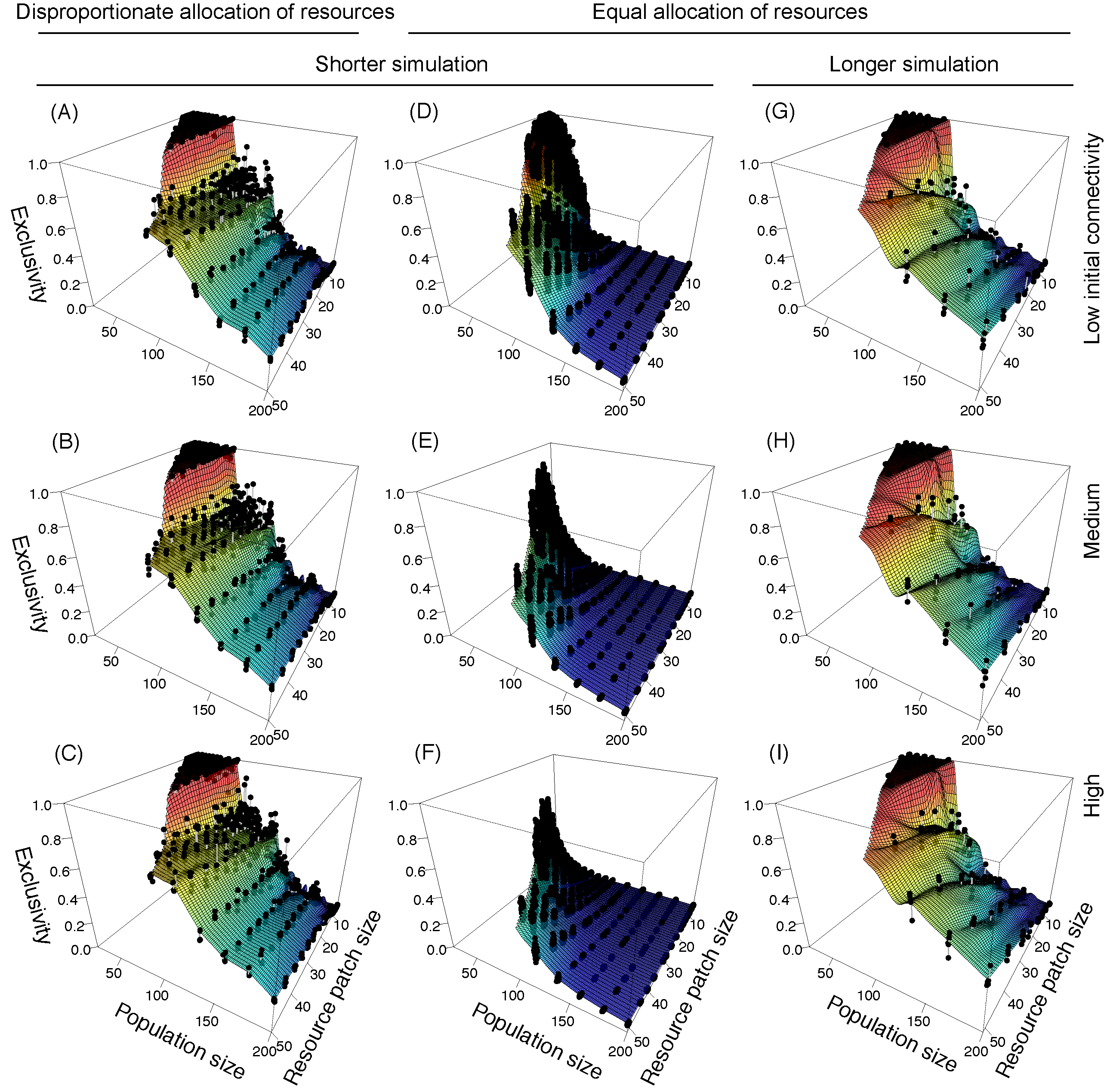


**Supplementary Figure S2:** The small effects of initial social network connectivity (rows) and the type of group-level resource share (columns) on the emergence of foraging groups in the baseline model. In all cases, the exclusivity (proportion of times individuals were members of the specialised foraging group) is highest in small population sizes (i.e. number of nodes in the network, *N*) and resource patch sizes (*R*), and decreases. Initial network connectivity varied between low (*T_prob_*=0.2, i.e. 20% of realized links randomly assigned to individuals), medium (*T_prob_*=0.5) and high (*T_prob_*=0.8). Type of group-level resource share varied between disproportionate allocation of resources (i.e. dependent on group size, in which larger groups outcompete smaller ones); and equal allocation of resources (i.e. independent of group size). The first two columns result from running the baseline model across the typical parameter space (see Sensitivity Analysis section in the main text: varying initial population size $N$ by increments of 5 ($P=\left\{ 2,7,12,\ldots,200 \right\}, N\in P$) and resource patch size $R$ by increments of 2 ($S=\left\{ 5,7,9,\ldots,51 \right\}, R\in S$), with 500 replicates run for generally short periods ($t=N\cdot5$ time steps). The results in the third column come from running the same model for longer periods ($t=500$ time steps for all parameter combinations) across a restricted parameter space ($P=\left\{ 20,30,40,50,70,100,140,170,200 \right\}, N\in P$; $S=\left\{ 5,9,13,17, 25,31,41,51 \right\}, R\in S$; fewer replicates) to show that under the scenario of equal allocation of the resource, the emergence of specialisation takes longer to establish.


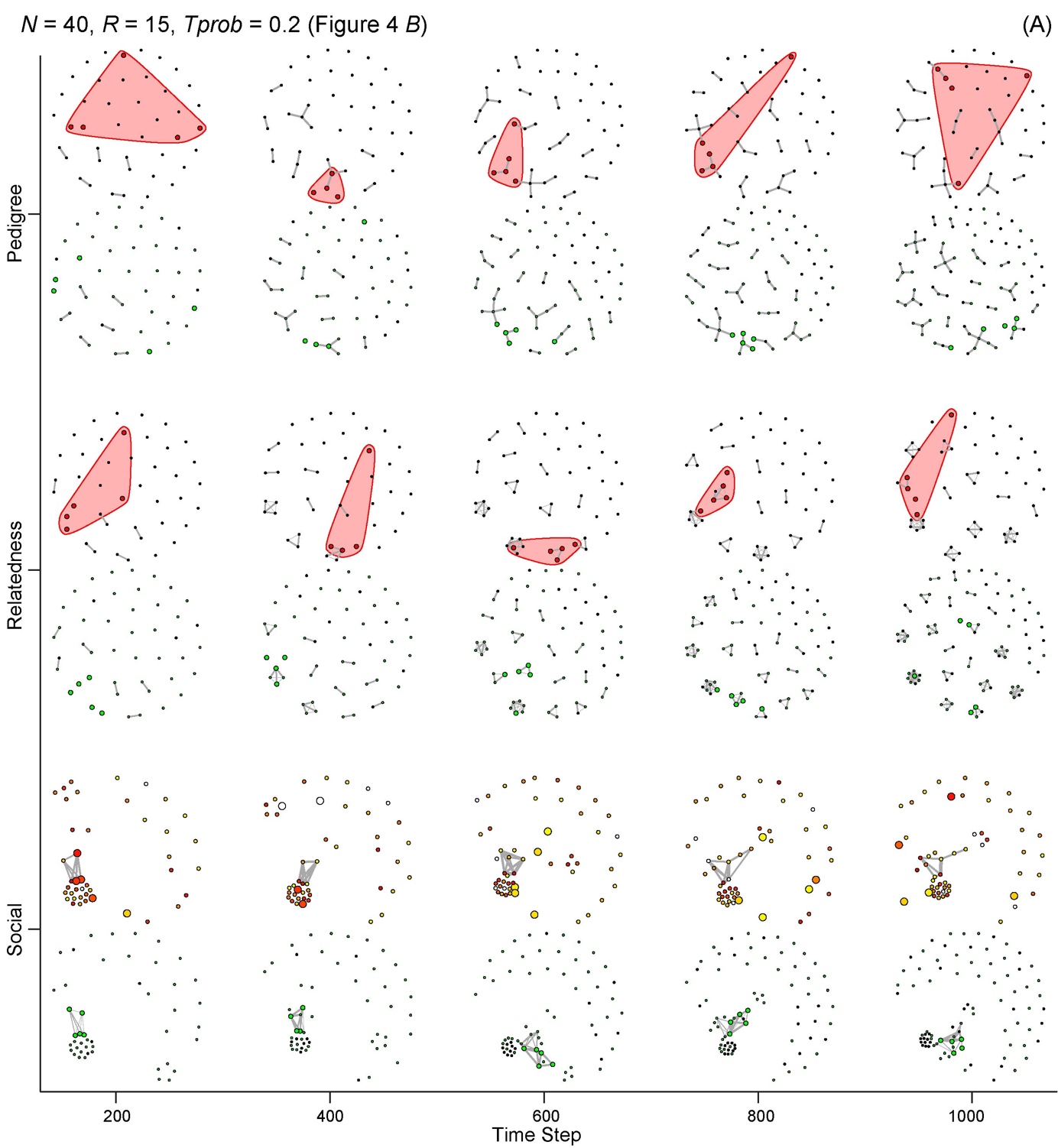


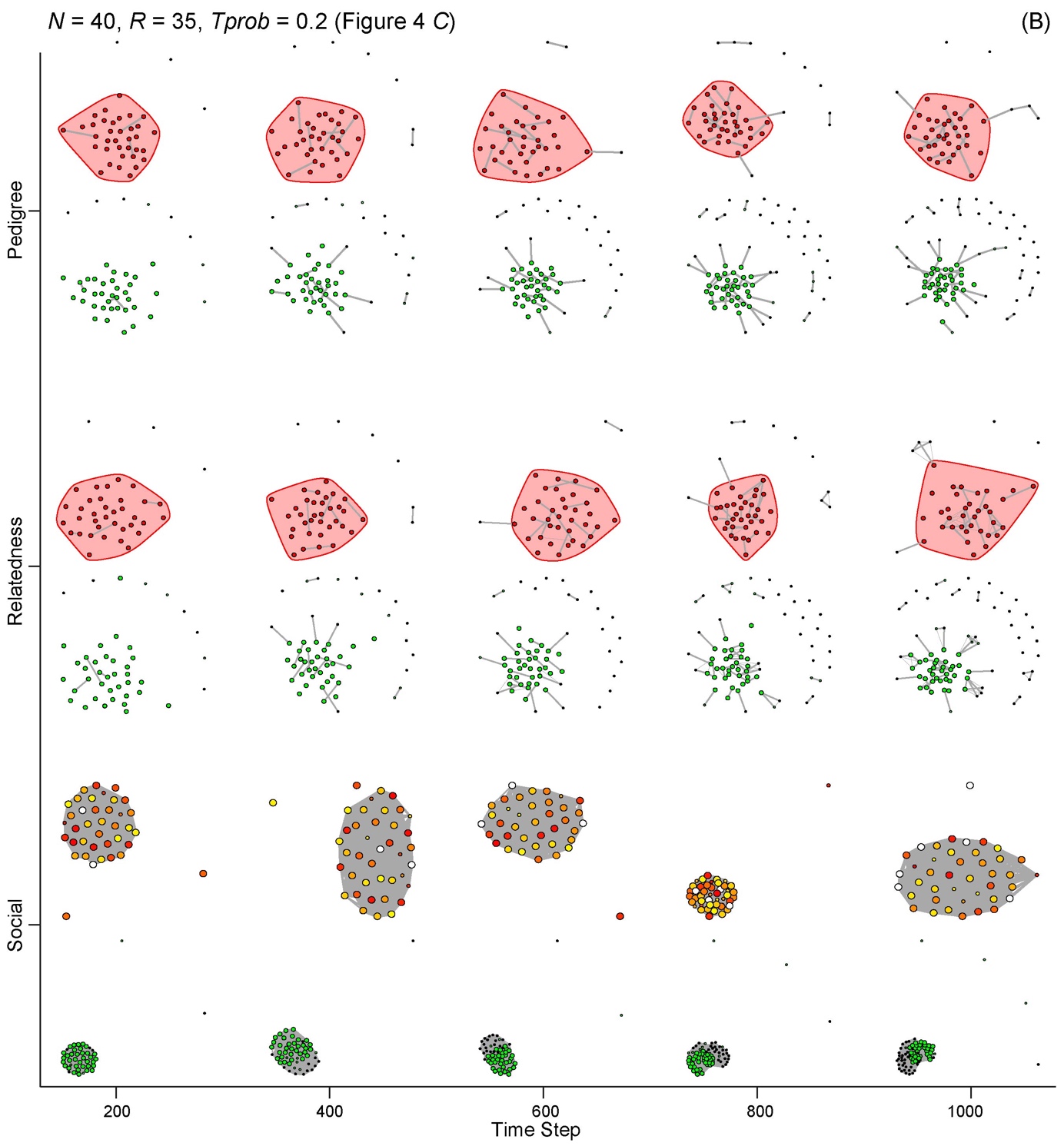


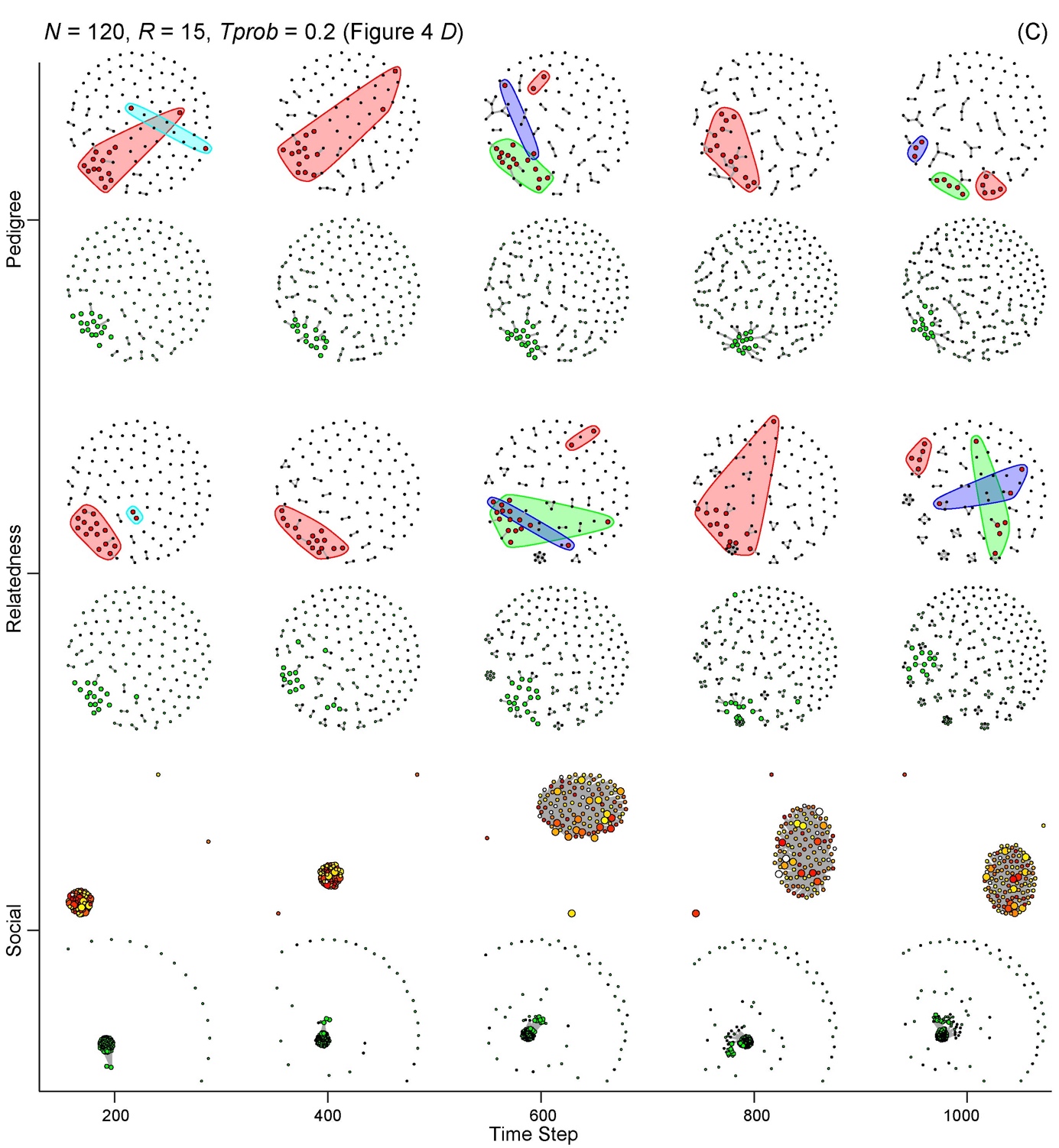


**
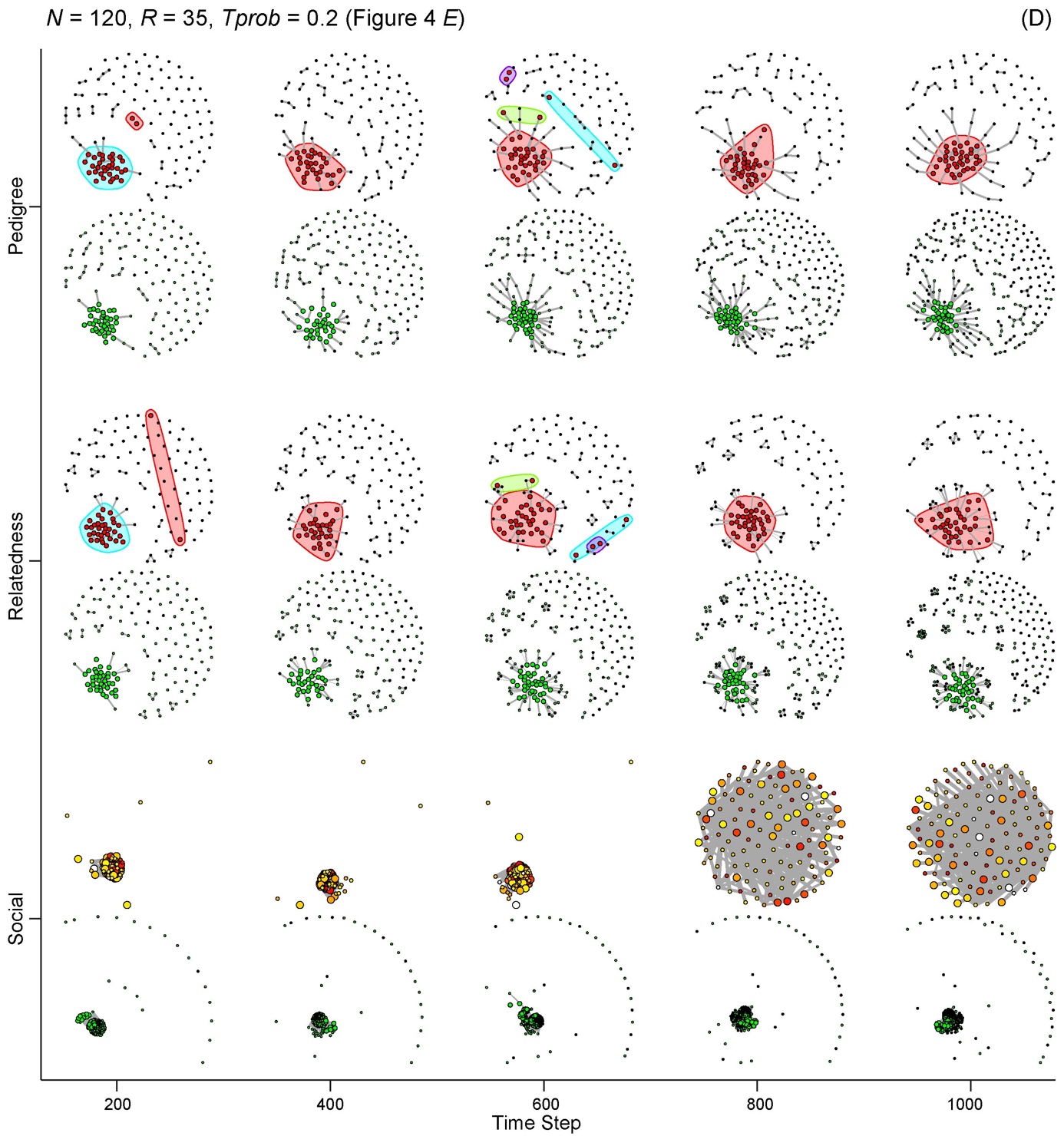
 Supplementary Figure S3:** Evolution of pedigree, relatedness and social relationships among all individuals throughout (in five snapshots of) the simulation time for the four corners of parameter space used in Fig. 1 and Fig. 3. (A) small population size, small resource patch size (*N*=40, *R*=15, *T_prob_=*0.2); (B) small population size, large resource patch size (*N*=40, *R*=35, *T_prob_*=0.2); (C) large population size, small resource patch size (*N*=120, *R*=15, *T_prob_=*0.2); (D) large population size, large resource patch size (*N*=120, *R*=35, $T_{\mathrm{prob}}$*T_prob_=*0.2). For each parameter combination, there are two sets of networks: the top set contain only alive agents at that given time step; the bottom set contain both alive (green nodes) and dead agents (black nodes). Within each set, the top row networks represent agents by nodes connected by binary links depicting parent-calf interactions; the mid row represent agents connected by weighted links whose thicknesses are proportional to their relatedness; and the bottom row represent agents connected by the proportion of times they were members of a foraging group (for better visualization, the links of these social networks whose weights <0.5 were filtered off). In all networks, all members of the current foraging groups are indicated by larger nodes. Coloured polygons indicate distinct foraging groups. Simulation was run for initial connectivity, *T_prob_*=0.2, and lasted for 1000 time steps.


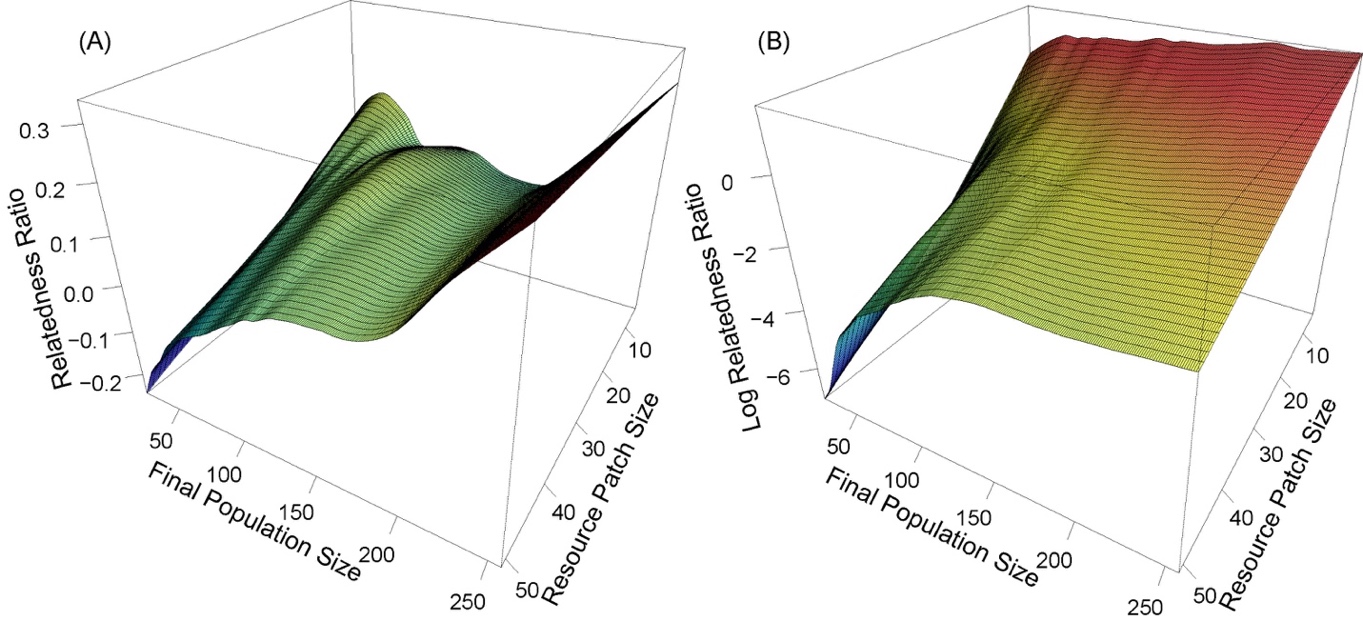


**Supplementary Figure S4:** Relatedness ratio at the end of the simulation. Relatedness at time step *t=*1000 is shown as a function of final population size *N* and resource patch size *R*. (A) Ratio of the relatedness ratio between members and non-members of specialised foraging groups. (B) Log of the relatedness ratio. Initial connectivity, *T_prob_*=0.5.
